# Triglyceride-glucose index as a predictor of one-year mortality in non-diabetic acute ischemic stroke

**DOI:** 10.3389/fendo.2025.1523787

**Published:** 2025-05-22

**Authors:** Shengyuan Wang, Xianjia Ning, Jun Tu, Jinghua Wang, Yu Zhao

**Affiliations:** ^1^ Department of Neurology, Chongqing General Hospital, Chongqing University, Chongqing, China; ^2^ Department of Neurology, Tianjin Medical University General Hospital, Tianjin, China; ^3^ Laboratory of Epidemiology, Tianjin Neurological Institute, Tianjin, China; ^4^ Tianjin Neurological Institute, Key Laboratory of Post-Neuroinjury Neuro-Repair and Regeneration in Central Nervous System, Ministry of Education and Tianjin City, Tianjin, China; ^5^ Center of Clinical Epidemiology, Shenzhen Third People’s Hospital and the Second Hospital Affiliated with the Southern University of Science and Technology, Shenzhen, Guangdong, China; ^6^ Department of Neurology, Shenzhen Third People’s Hospital and the Second Hospital Affiliated with the Southern University of Science and Technology, Shenzhen, Guangdong, China

**Keywords:** triglyceride-glucose index, ischemic stroke, non-diabetic, mortality, recurrence

## Abstract

**Background:**

Acute ischemic stroke (AIS) is a leading cause of morbidity and mortality, and identifying reliable prognostic markers is crucial for improving outcomes. The triglyceride-glucose (TyG) index, a surrogate marker for insulin resistance, has been associated with adverse cardiovascular outcomes. However, its role in predicting stroke prognosis, particularly in non-diabetic patients, remains unclear. This study aimed to explore the association between the TyG index and one-year outcomes, including mortality, recurrence, and adverse functional outcomes, in non-diabetic IS patients.

**Methods:**

This prospective cohort study included AIS patients without diabetes from multiple hospitals. Baseline data, including the TyG index, were collected at admission, and patients were followed for one year. The primary outcomes were all-cause mortality, stroke recurrence, and adverse functional outcomes, defined as modified Rankin Scale (mRS) >2. Multivariate logistic regression and subgroup analyses were conducted to assess the predictive value of the TyG index for these outcomes.

**Results:**

Among the study population, 5.9% died within one year. The TyG index and its quartiles were significantly associated with one-year mortality, even after adjusting for confounding factors. Patients in the highest TyG quartile (Q4: TyG ≥ 8.9002) had a 3.72-fold higher risk of mortality compared to those in the lowest quartile (P = 0.013). Subgroup analysis showed that the TyG index was a stronger predictor of mortality in men and non-atrial fibrillation patients. Although the TyG index was not significantly associated with stroke recurrence or adverse functional outcomes in the overall cohort, it acted as a protective factor for recurrence in younger patients (< 65 years).

**Conclusion:**

The TyG index is an independent predictor of one-year mortality in non-diabetic IS patients and may aid in risk stratification, particularly in men and younger patients. Its potential role in predicting recurrence and functional outcomes warrants further investigation.

## 1 Introduction

Stroke is primarily characterized by neurological deficits and is caused by acute focal injury to the central nervous system (CNS) due to vascular events, including ischemic stroke (IS) and hemorrhagic stroke (HS). It remains one of the leading causes of disability and mortality globally (1). From 1990 to 2019, the absolute number of stroke cases increased by 70%, with a corresponding 85% rise in prevalence and a 43% increase in stroke-related deaths (2). Each year in the United States, approximately 795,000 people experience a stroke, with 87% of these being ischemic, and 185,000 are recurrent strokes (3). In China, stroke is the second leading cause of death among rural residents and the third among urban residents. The standardized prevalence of stroke among middle-aged and elderly individuals is 2610 per 100,000, with a recurrence rate of 17% within one year (4). From 2010 to 2021, stroke was the leading cause of disability-adjusted life years (DALYs) globally, particularly in China, where it ranks first among the top 10 neurological disorders with the highest age-standardized DALYs (5, 6). The global economic burden of stroke, considering both direct (treatment and rehabilitation) and indirect (loss of productivity) costs, exceeds $891 billion annually (7). Over the past three decades, stroke incidence and burden in China have escalated, especially in rural areas, with North and Central China bearing the heaviest burden (8).

Multiple studies have identified insulin resistance (IR) as a critical risk factor for stroke (9). IR can enhance platelet adhesion, activation, and aggregation, while also inducing hemodynamic disturbances that increase the likelihood of vascular events (10). It has been shown that IR negatively impacts stroke prognosis, irrespective of the patient’s diabetic status (11). The TyG index, a parameter derived from fasting blood glucose and triglyceride levels, has been recognized as a reliable marker of insulin resistance (12, 13). Meta-analyses have demonstrated that a high TyG index is significantly associated with stroke recurrence and increased mortality (14). Retrospective studies have further highlighted the association of the TyG index with stroke death and short-term adverse functional outcomes in the general population (15, 16). Additionally, in critically ill IS patients, the TyG index has been linked to increased risk of hospitalization and ICU mortality (17, 18). Long-term prospective studies in both the American population and rural China have indicated that the TyG index independently predicts stroke progression (19, 20), while other studies have found its association with first-time stroke in young adults in China (21). Short-term studies have reported significant correlations between TyG index and stroke recurrence, all-cause mortality, and neurological deterioration (22), especially among elderly IS patients, female patients, and those with mild IS and hypertension (23–25). However, the predictive value of the TyG index in non-diabetic acute ischemic stroke (AIS) patients remains inconclusive (26).

Most research on the TyG index and stroke prognosis has focused on short-term outcomes and general population studies. Given that age is a non-modifiable stroke risk factor, earlier intervention on modifiable factors such as hypertension, diabetes, and dyslipidemia is essential (4). However, there is a paucity of research exploring TyG’s impact on stroke prognosis when populations are stratified by such risk factors, particularly in non-diabetic patients. While studies indicate a significant relationship between the TyG index and stroke risk (27), its role in predicting long-term outcomes remains unclear. Given that stroke is a leading cause of death in China, especially in rural areas, timely prediction of stroke prognosis and early intervention are crucial.

The purpose of this study is to evaluate the influence of the TyG index on the prognosis of acute stroke patients without diabetes through a prospective cohort study.

## 2 Methods

### 2.1 Study population

This prospective cohort study conducted between January 2021 and July 2023, enrolled acute ischemic stroke patients without diabetes. All patients were diagnosed according to the World Health Organization criteria, and diagnoses were confirmed using brain computed tomography or magnetic resonance imaging.

The study was approved by the Medical Ethics Committee of Shenzhen Third People’s Hospital (KY2025-082-01) and adhered to the ethical guidelines of the Declaration of Helsinki. Informed consent was obtained from all participants.

### 2.2 Inclusion and exclusion criteria

Inclusion criteria included that (1) Patients aged 18 years or older; (2)patients diagnosed with acute ischemic stroke, confirmed by clinical evaluation and neuroimaging; (3) patients without a diagnosis of diabetes, as determined by medical history, fasting plasma glucose (FPG) levels < 7.0 mmol/L, and HbA1c < 6.5%; (4) patients with complete baseline data on fasting triglyceride (TG) and fasting blood glucose (FBG) levels for calculating the TyG index; (5) patients or their legal representatives provided written informed consent to participate in the study.

Exclusion criteria included that (1) patients with a prior diagnosis of diabetes or those with FPG ≥ 7.0 mmol/L or HbA1c ≥ 6.5%; This exclusion was intended to minimize confounding, as diabetes is an established strong risk factor for stroke prognosis and could independently influence TyG index levels, thus complicating the assessment of TyG’s prognostic value in a non-diabetic population. (2) patients with hemorrhagic stroke or transient ischemic attack (TIA); (3) patients with incomplete baseline data, including missing TG or FBG values.The missing data were primarily due to variations in clinical practice among participating centers, where fasting blood samples were not consistently collected at admission, particularly in patients with mild symptoms or early discharges; (4) patients with severe systemic diseases such as advanced liver disease, end-stage renal disease, or malignancy, which could significantly affect prognosis; and (5) patients who were unwilling or unable to complete the one-year follow-up or withdrew consent during the study period.

Trained neurologists collected detailed patient information, including stroke subtype, stroke severity, previous medical history, lifestyle factors, and 1-year post-stroke outcomes.

### 2.3 Data collection

Sociodemographic and clinical information, including name, gender, age, lifestyle, and history of diabetes, hypertension, and atrial fibrillation, was gathered through face-to-face interviews conducted by trained professional researchers. Laboratory tests included FBG, total cholesterol (TC), TG, high-density lipoprotein (HDL), low-density lipoprotein (LDL), and homocysteine (HCY) levels. Systolic blood pressure (SBP) and diastolic blood pressure (DBP) were measured using an automated sphygmomanometer. Patients were asked to rest for 15 minutes before measurements, and blood pressure was taken in both arms, with repeated measurements every two minutes. The average value was recorded.

### 2.4 Definition and grouping

Smoking was defined in accordance with the World Health Organization (WHO) recommendations, as continuous or cumulative smoking for six months or more in one’s life (28, 29). Alcohol consumption, classified as heavy drinking, was defined as ≥60g of pure alcohol per day for men and ≥ 40g for women. Sedentary behavior was defined as any waking activity characterized by sitting, reclining, or lying posture with ≤1.5 metabolic equivalents (MET) (30). This equates to more than 8 hours of sedentary behavior per day for five or more days per week. Hypertension was defined as SBP ≥ 140 mmHg and/or DBP ≥ 90 mmHg or a self-reported history of hypertension and use of antihypertensive medication (31). Diabetes was diagnosed based on glycated hemoglobin ≥ 6.5%, FPG ≥ 126 mg/dL (7.0 mmol/L), 2-hour plasma glucose (PG) ≥ 200 mg/dL during an oral glucose tolerance test (OGTT), or a history of diabetes and hypoglycemic medication use (32). Hyperlipidemia was classified as hypercholesterolemia (total cholesterol ≥ 6.2 mmol/L), hypertriglyceridemia (triglyceride ≥ 2.3 mmol/L), or dyslipidemia, including LDL ≥ 4.1 mmol/L (33). Hyperhomocysteinemia was defined as a blood homocysteine level exceeding 15 μmol/L (34). The TyG index was calculated using the standardized formula: ln (fasting triglyceride [mg/dL] × fasting blood glucose [mg/dL]/2) (35). Participants were then grouped into quartiles based on their TyG index values.

### 2.5 Outcome Definitions and Assessments

The primary outcomes of this study were one-year mortality, stroke recurrence, and adverse functional outcomes. One-year mortality was defined as all-cause death occurring within one year of the initial stroke event. Stroke recurrence was defined as any new ischemic or hemorrhagic stroke occurring within one year after the index stroke, confirmed by clinical symptoms and imaging studies. Adverse functional outcomes were assessed using mRS, with poor outcomes defined as an mRS score greater than 2, indicating moderate to severe disability (36). The mRS assessments were conducted during follow-up visits by trained clinicians who were blinded to the baseline TyG index values. All outcomes were tracked through hospital records, follow-up appointments, and telephone interviews with patients or their family members. These outcomes were analyzed to determine the predictive value of the TyG index in relation to stroke prognosis at one year. Loss to follow-up was defined as cases where no further follow-up data could be obtained through existing contact methods (telephone, address, and emergency contacts).

### 2.6 Statistical analysis

Continuous variables were expressed as mean ± standard deviation (SD) or median (P25–P75) and compared using the T-test or Mann-Whitney U test, as appropriate. Categorical variables were expressed as frequency and percentage, and comparisons were made using the chi-square test. Multivariate logistic regression analysis was employed to examine the relationship between the TyG index and stroke outcomes, including mortality, recurrence, and adverse outcomes at one-year follow-up. Variables with significant associations in univariate analysis were included in the multivariate analysis. The results were reported as relative risk (RR) with 95% confidence intervals (CI).

Subgroup analysis was performed to further explore the relationship between TyG index and stroke outcomes in different patient subgroups, based on variables identified in the univariate analysis. Statistical significance was set at P < 0.05. All statistical analyses were conducted using SPSS (version 2.7) and GraphPad Prism (version 10.2.3).

## 3 Result

**Figure 1:**
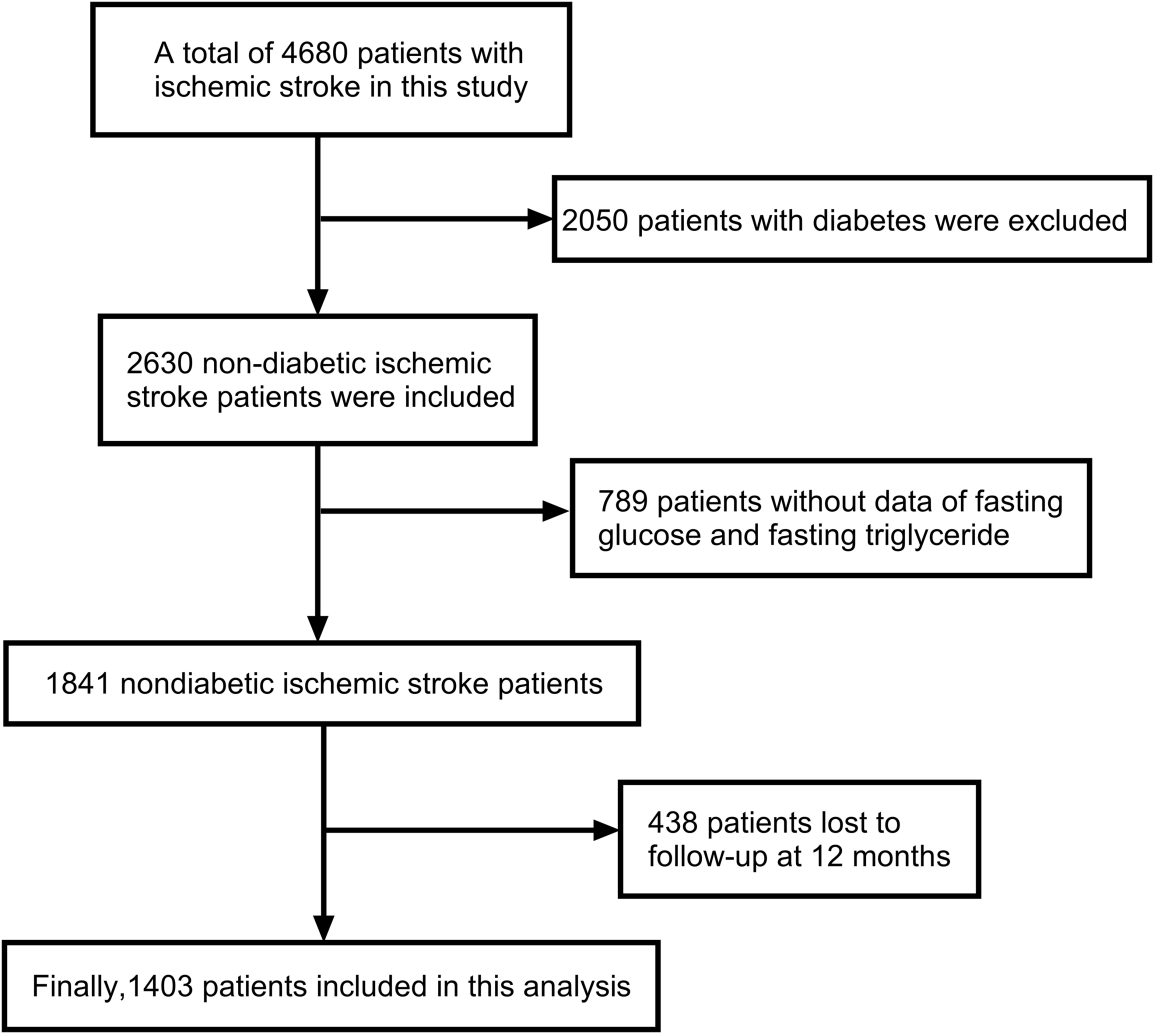
Flow chat of participants selection. Image showed that 4680 patients with ischemic stroke were included in this study, from which 2050 patients with diabetes were excluded. Subsequently, 2630 non-diabetic ischemic stroke patients were included, but 789 patients were excluded due to the lack of data on fasting glucose and fasting triglyceride. During the 12-month follow-up, another 438 patients were lost to follow-up. Finally, 1403 patients were included in this analysis.

The study included 1,841 non-diabetic ischemic stroke patients, with complete baseline fasting blood glucose and triglyceride data, were selected for one-year follow-up. After excluding 438 patients lost to follow-up, 1,403 participants remained in the study (**Figure 1**).

### 3.1 Demographic characteristics

Of these 1,403 patients, there were 998 males (71.1%) and 405 females (28.9%). The average age was 62.99 ± 11.70 years. The mean admission NIHSS and BI scores were 5.96 ± 5.78 and 64.96 ± 29.66, respectively. The mean levels of TC, TG, HDL, and LDL were 4.82 ± 1.04 mmol/L, 1.48 ± 0.98 mmol/L, 1.09 ± 0.31 mmol/L, and 2.99 ± 0.84 mmol/L, respectively. DBP averaged 86.48 ± 14.29 mmHg. Key laboratory findings included creatinine (68.87 ± 23.97 μmol/L), urea nitrogen (5.43 ± 1.90 mmol/L), albumin (38.98 ± 3.70 g/L), and globulin (28.81 ± 4.43 g/L). The average TyG index was 8.58 ± 0.53 (**Table 1**).

**Table 1:** Baseline demographic characteristics.

Characteristics	Man	Woman	Total
Total, n (%)	998 (71.1)	405 (28.9)	1403 (100)
Age, years old	61.64 (11.56)	66.31 (11.41)	62.99 (11.70)
Age group, n (%)			
< 45 years old	57 (5.7)	9 (2.2)	66 (4.7)
45-64 years old	549 (55.0)	154 (38.0)	703 (50.1)
65-79 years old	326 (32.7)	194 (47.9)	520 (37.1)
≥80 years old	66 (6.6)	48 (11.9)	114 (8.1)
Admission NIHSS scores	5.54 (5.55)	7.01 (6.20)	5.96 (5.78)
Admission BI scores	67.35 (28.88)	59.06 (30.76)	64.96 (29.66)
Atrial fibrillation, n (%)			
No	937 (93.9)	350 (86.4)	1287 (91.7)
Yes	61 (6.1)	55 (13.6)	116 (8.3)
Hyperhomocysteinemia, n (%)			
No	610 (61.1)	336 (83.0)	946 (67.4)
Yes	388 (38.9)	69 (17.0)	457 (32.6)
Smoking history, n (%)			
No	460 (46.1)	342 (84.4)	802 (57.2)
Yes	538 (53.9)	63 (15.6)	601 (42.8)
Drinking history, n (%)			
No	678 (67.9)	397 (98.0)	1075 (76.6)
Yes	320 (32.1)	8 (2.0)	328 (23.4)
Sedentary lifestyle, n (%)			
No	935 (93.7)	360 (88.9)	1295 (92.3)
Yes	63 (6.3)	45 (11.1)	108 (7.7)
Cerebral artery stenosis, n (%)			
No	773 (77.5)	336 (83.0)	1109 (79.0)
Yes	225 (22.5)	69 (17.0)	294 (21.0)
Hypertension, n (%)			
No	160 (16.0)	46 (11.4)	206 (14.7)
Yes	838 (84.0)	359 (88.6)	1197 (85.3)
Hyperlipidemia, n (%)			
No	618 (61.9)	245 (60.5)	863 (61.5)
Yes	380 (38.1)	160 (39.5)	540 (38.5)
Total cholesterol TC, mmol/L	4.69 (1.01)	5.16 (1.03)	4.82 (1.04)
Triglyceride TG, mmol/L	1.49 (0.96)	1.45 (1.02)	1.48 (0.98)
High density lipoprotein HDL, mmol/L	1.05 (0.30)	1.20 (0.32)	1.09 (0.31)
Low density lipoprotein LDL, mmol/L	2.91 (0.82)	3.19 (0.84)	2.99 (0.84)
Fasting blood glucose FBG, mmol/L	5.20 (0.70)	5.31 (0.71)	5.23 (0.71)
Systolic blood pressure SBP, mmHg^*^	154.01 (24.47)	158.79 (27.93)	155.38 (25.60)
Diastolic pressure DBP, mmHg^*^	83.84 (14.25)	83.84 (14.05)	86.48 (14.29)
Creatinine, μmol/L^*^	73.51 (24.80)	57.02 (16.63)	68.87 (23.97)
Urea nitrogen BUN, mmol/L^*^	5.53 (1.94)	5.17 (1.78)	5.43 (1.90)
Albumin, g/L^*^	39.15 (3.59)	38.56 (3.94)	38.98 (3.70)
Globulin, g/L^*^	28.27 (4.32)	30.17 (4.43)	28.81 (4.43)
Alanine transaminase ALT, U/L^*^	26.96 (30.71)	21.58 (21.50)	25.45 (28.52)
Aspertate aminotransferase AST, U/L^*^	23.99 (19.21)	23.45 (13.56)	23.83 (17.77)
Direct bilirubin CB, μmol/L^*^	5.25 (2.95)	4.30 (2.28)	4.98 (2.81)
Indirect bilirubin UCB, μmol/L^*^	9.72 (5.20)	8.76 (4.45)	9.45 (5.02)
White blood cell count, ×10^9/L^*^	7.71 (2.43)	7.11 (2.55)	7.54 (2.48)
Red blood cell count, ×10^12/L^*^	4.68 (0.60)	4.30 (0.51)	4.57 (0.60)
Platelet count, ×10^9/L ^*^	210.14 (61.08)	231.06 (73.68)	216.15 (65.61)
Hemoglobin concentration, g/L^*^	147.29 (16.39)	130.71 (73.68)	142.53 (17.56)
TyG index	8.58 (0.53)	8.58 (0.51)	8.58 (0.53)
TyG index quartile			
Q1	262 (26.3)	88 (21.7)	350 (24.9)
Q2	244 (24.4)	107 (26.4)	351 (25.0)
Q3	239 (23.9)	113 (27.9)	352 (25.1)
Q4	253 (25.4)	97 (24.0)	350 (24.9)

(1) The results of continuous variables are expressed as mean (SD).

(2) ^*^ It means that there are missing values, including 220 cases of SBP missing in hospital, 221 cases of DBP missing in hospital, 134 cases of creatinine missing, 136 cases of urea nitrogen missing, 141 cases of albumin missing, 143 cases of globulin missing, 143 cases of ALT missing, 23 cases of AST missing, 145 cases of direct bilirubin missing, 145 cases of indirect bilirubin missing, 16 cases of white blood cells and hemoglobin concentration missing, and 18 cases of red blood cells and platelets missing.

To assess the potential impact of loss to follow-up on the study results, we conducted a supplementary analysis comparing the baseline characteristics between the followed and lost-to-follow-up groups (**Supplementary Table 1**). The results showed no statistically significant differences (all P > 0.05), suggesting that the missing data are likely to be missing at random and do not introduce systematic bias.

### 3.2 Univariate analysis of factors influencing one-year outcomes

Among the 1,403 patients followed for one year, 83 died, resulting in a mortality rate of 5.9%. Univariate analysis identified several factors significantly associated with one-year mortality, including age, admission NIHSS and BI scores, atrial fibrillation, hyperhomocysteinemia, smoking, alcohol consumption, sedentary lifestyle, triglycerides, fasting blood glucose, urea nitrogen, albumin, globulin, AST, bilirubin (direct and indirect), white and red blood cell counts, platelets, hemoglobin, and the TyG index and its quartiles (P < 0.05). For stroke recurrence, 363 cases were observed among the 1,353 patients (26.8%). Factors significantly related to recurrence included gender, age, admission NIHSS and BI scores, atrial fibrillation, hyperhomocysteinemia, alcohol use, sedentary lifestyle, albumin, and LDL levels (P < 0.05). In terms of adverse functional outcomes (mRS > 2), observed in 342 survivors (25.9%), significant factors included gender, age, admission NIHSS and BI scores, hyperhomocysteinemia, sedentary lifestyle, LDL, and indirect bilirubin (P < 0.05) (**Table 2**).

**Table 2:** Univariate analysis of risk of death, recurrence risk and adverse outcomes among survivors one year after stroke.

Characteristics	Death			Recurrence			Adverse outcomes		
	Yes	No	P value	Yes	No	P value	Yes	No	P value
Total, n (%)	83 (5.9)	1320 (94.1)		363 (26.8)	990 (73.2)		342 (25.9)	978 (74.1)	
Gender, n (%)			0.992			0.011			0.011
Man	59 (71.1)	939 (71.1)		239 (65.8)	722 (72.9)		225 (65.8)	714 (73.0)	
Woman	24 (28.9)	381 (28.9)		124 (34.2)	268 (27.1)		117 (34.2)	264 (27.0)	
Age, years old	72.64 (10.88)	62.38 (11.49)	<0.001	65.21 (12.03)	61.61 (11.17)	<0.001	65.04 (12.08)	61.45 (11.13)	<0.001
Age group, n (%)			<0.001			<0.001			<0.001
< 45 years old	0 (0.0)	66 (100.0)		13 (3.6)	53 (5.4)		13 (3.8)	53 (5.4)	
45-64 years old	20 (24.1)	683 (51.7)		148 (40.8)	544 (54.9)		140 (40.9)	543 (55.5)	
65-79 years old	35 (42.2)	485 (36.7)		163 (44.9)	339 (34.2)		154 (45.0)	331 (33.8)	
≥80 years old	28 (33.7)	86 (6.5)		39 (10.7)	54 (5.5)		35 (10.2)	51 (5.2)	
Admission NIHSS score, M (P25, P75)	14 (8-22)	4 (2-7)	<0.001	5 (2-9)	4 (2-7)	<0.001	5 (2-9)	4 (2-7)	<0.001
BI score of admission, M (P25, P75)	15 (0-45)	70 (50-95)	<0.001	60 (35-90)	70 (50-95)	<0.001	60 (40-90)	70 (50-95)	<0.001
Atrial fibrillation, n (%)			<0.001			0.013			0.056
No	57 (68.7)	1230 (93.2)		325 (89.5)	926 (93.5)		311 (90.9)	919 (94.0)	
Yes	26 (31.3)	90 (6.8)		38 (10.5)	64 (6.5)		31 (9.1)	59 (6.0)	
Hyperhomocysteinemia, n (%)			0.016			0.045			0.041
No	46 (55.4)	900 (68.2)		231 (63.6)	687 (69.4)		218 (63.7)	682 (69.7)	
Yes	37 (44.6)	420 (31.8)		132 (36.4)	303 (30.6)		124 (36.3)	296 (30.3)	
Smoking history, n (%)			0.002			0.146			0.128
No	61 (73.5)	741 (56.1)		217 (59.8)	548 (55.4)		204 (59.6)	537 (54.9)	
Yes	22 (26.5)	579 (43.9)		146 (40.2)	442 (44.6)		138 (40.4)	441 (45.1)	
Drinking history, n (%)			<0.001			0.020			0.054
No	76 (91.6)	999 (75.7)		293 (80.7)	739 (74.6)		272 (79.5)	727 (74.3)	
Yes	7 (8.4)	321 (24.3)		70 (19.3)	251 (25.4)		70 (20.5)	251 (25.7)	
Sedentary lifestyle, n (%)			0.017			<0.001			<0.001
No	71 (85.5)	1224 (92.7)		319 (87.9)	933 (94.2)		300 (87.7)	924 (94.5)	
Yes	12 (14.5)	96 (7.3)		44 (12.1)	57 (5.8)		42 (12.3)	54 (5.5)	
Cerebral artery stenosis, n (%)			0.506			0.764			0.913
No	68 (81.9)	1041 (78.9)		284 (78.2)	782 (79.0)		269 (78.7)	772 (78.9)	
Yes	15 (18.1)	279 (21.1)		79 (21.8)	208 (21.0)		73 (21.3)	206 (21.1)	
Hypertension, n (%)			0.368			0.842			0.931
No	15 (18.1)	191 (15.4)		54 (14.9)	143 (14.4)		49 (14.3)	142 (14.5)	
Yes	68 (81.9)	1129 (85.5)		309 (85.1)	847 (85.6)		293 (85.7)	836 (85.5)	
Hyperlipidemia, n (%)			0.826			0.240			0.202
No	52 (62.7)	811 (61.4)		232 (63.9)	598 (60.4)		220 (64.3)	591 (60.4)	
Yes	31 (37.3)	509 (38.6)		131 (36.1)	392 (39.6)		122 (35.7)	387 (39.6)	
Total cholesterol TC, mmol/L	4.67 (1.16)	4.83 (1.03)	0.158	4.74 (1.04)	4.86 (1.03)	0.056	4.75 (1.04)	4.86 (1.03)	0.081
Triglyceride TG, mmol/L	1.19 (0.94)	1.49 (0.98)	0.006	1.43 (1.04)	1.51 (0.96)	0.208	1.44 (1.01)	1.51 (0.97)	0.210
High density lipoprotein HDL, mmol/L	1.17 (0.61)	1.09 (0.28)	0.222	1.10 (0.31)	1.09 (0.27)	0.541	1.10 (0.31)	1.08 (0.27)	0.451
Low density lipoprotein LDL, mmol/L	2.90 (0.96)	2.99 (0.83)	0.391	2.91 (0.87)	3.02 (0.82)	0.026	2.91 (0.86)	3.02 (0.82)	0.039
Fasting blood glucose FBG, mmol/L	5.48 (0.74)	5.22 (0.70)	0.001	5.26 (0.69)	5.21 (0.70)	0.230	5.25 (0.70)	5.21 (0.70)	0.353
Systolic blood pressure SBP, mmHg^*^	155.35 (33.72)	155.38 (25.05)	0.993	154.15 (28.49)	155.64 (24.04)	0.407	155.00 (27.59)	155.53 (24.05)	0.768
Diastolic pressure DBP, mmHg^*^	85.68 (15.85)	86.53 (14.20)	0.847	86.19 (15.83)	86.69 (13.51)	0.616	86.18 (15.96)	86.66 (13.48)	0.637
Creatinine, μmol/L^*^	79.02 (56.07)	68.18 (20.30)	0.099	69.49 (26.23)	68.48 (22.34)	0.506	69.42 (26.42)	67.83 (17.60)	0.237
Urea nitrogen BUN, mmol/L^*^	6.58 (3.62)	5.35 (1.71)	0.005	5.52 (1.99)	5.33 (1.72)	0.098	5.51 (2.02)	5.30 (1.59)	0.072
Albumin ABL, g/L^*^	35.87 (4.79)	39.18 (3.53)	<0.001	38.69 (3.62)	39.23 (3.58)	0.019	38.86 (3.44)	39.29 (3.56)	0.073
Globulin, g/L^*^	30.31 (5.01)	28.71 (4.38)	0.002	28.71 (4.150	28.77 (4.50)	0.821	28.62 (4.13)	28.74 (4.46)	0.682
Alanine transaminase ALT, U/L^*^	24.28 (22.14)	25.53 (28.88)	0.717	25.65 (40.81)	25.24 (22.69)	0.825	26.09 (41.97)	25.33 (22.81)	0.693
Aspertate aminotransferase AST, U/L^*^	31.41 (25.56)	23.37 (17.08)	0.007	23.70 (20.52)	23.32 (15.46)	0.723	23.62 (20.97)	23.28 (15.50)	0.752
Direct bilirubin CB, μmol/L^*^	6.92 (3.43)	4.86 (2.73)	<0.001	4.85 (2.57)	4.94 (2.81)	0.607	4.74 (2.52)	4.90 (2.79)	0.376
Indirect bilirubin UCB, μmol/L^*^	11.75 (5.43)	9.31 (4.96)	<0.001	8.98 (4.26)	9.55 (5.24)	0.084	8.76 (4.10)	9.49 (5.21)	0.013
White blood cell count, ×10^9/L^*^	9.24 (3.63)	7.43 (2.35)	<0.001	7.62 (2.52)	7.41 (2.37)	0.178	7.51 (2.34)	7.40 (2.36)	0.435
Red blood cell count, ×10^12/L^*^	4.40 (0.69)	4.58 (0.59)	0.008	4.55 (0.56)	4.59 (0.60)	0.307	4.56 (0.56)	4.59 (0.60)	0.351
Platelet count, ×10^9/L ^*^	195.01 (82.85)	217.48 (64.18)	0.018	217.95 (69.78)	216.62 (63.09)	0.752	218.26 (67.36)	217.21 (63.05)	0.801
Hemoglobin concentration, g/L^*^	136.67 (21.73)	142.90 (17.21)	0.013	141.55 (16.96)	143.20 (17.37)	0.120	141.67 (17.04)	143.33 (17.26)	0.125
TyG index	8.37 (0.59)	8.59 (0.52)	<0.001	8.54 (0.54)	8.60 (0.52)	0.063	8.55 (0.53)	8.60 (0.52)	0.090
TyG index quartile			0.007			0.118			0.076
Q1	33 (39.8)	317 (24.0)		99 (27.3)	231 (23.3)		92 (26.9)	225 (23.0)	
Q2	21 (25.3)	330 (25.0)		82 (22.6)	259 (26.2)		74 (21.6)	256 (26.2)	
Q3	16 (19.3)	336 (25.5)		101 (27.8)	241 (24.3)		98 (28.7)	238 (24.3)	
Q4	13 (15.7)	337 (25.5)		81 (22.3)	259 (26.2)		78 (22.8)	259 (26.5)	

(1) The results of continuous variables are expressed as mean (SD) and median (P25-P75).

(2)^*^ indicates that there are missing values, which are the same as the baseline data in risk of death.Recurrence risk missed values, including 211 cases of SBP deletion, 212 cases of DBP deletion, 129 cases of urea nitrogen deletion, 127 cases of creatinine deletion, 134 cases of albumin deletion, 136 cases of globulin deletion, 138 cases of direct bilirubin deletion, 138 cases of indirect bilirubin deletion, 136 cases of ALT deletion, 21 cases of AST deletion, 15 cases of white blood cell count deletion and hemoglobin concentration deletion,17 cases of red blood cell count and platelet count deletion. Adverse outcomes missed values, including 203 cases of SBP deletion, 204 cases of DBP deletion, 128 cases of urea nitrogen deletion, 126 cases of creatinine deletion, 132 cases of albumin deletion, 134 cases of globulin deletion, 136 cases of direct bilirubin deletion, 136 cases of indirect bilirubin deletion, 134 cases of ALT deletion, 20 cases of AST deletion, 15 cases of white blood cell count deletion and hemoglobin concentration deletion ,17 cases of red blood cell count and platelet count deletion.

### 3.3 Multivariate analysis of the relationship between TyG index and one-year outcomes

Multivariate analysis revealed that increasing age (RR: 1.05, 95% CI: 1.02, 1.09, P = 0.002), admission NIHSS score (RR: 1.10, P = 0.005), white blood cell count (RR: 1.12, P = 0.049), and AST (RR: 1.03, P = 0.021) were independent risk factors for one-year mortality, while albumin (RR: 0.87, P = 0.002) was protective. The TyG index was a significant predictor of mortality, with patients in the highest quartile (TyG ≥ 0.002) having a 3.72-fold higher risk compared to those in the lowest quartile (TyG < 8.2321, P = 0.013). For recurrence, higher age (P = 0.002) and ALT (RR: 1.01, P = 0.030) increased risk, while higher BI scores (P = 0.003) and LDL (RR: 0.64, P = 0.020) were protective. However, no significant relationship was found between the TyG index and stroke recurrence in multivariate analysis. Similarly, for adverse outcomes, age (P = 0.002), sedentary lifestyle (P = 0.048), and ALT (P = 0.022) increased risk, while LDL and UCB were protective, with no significant association found between TyG index and adverse outcomes in multivariate analysis (**Table 3**).

**Table 3:** Multivariate analysis of risk of death, recurrence risk and adverse outcomes after stroke for one year.

Characteristics	Reference	RR (95%CI)	P value
Risk of death			
TyG index		2.17 (1.09, 4.34)	0.028
TyG index quartile	Q1		
Q2		1.66 (0.73, 3.77)	0.223
Q3		1.48 (0.60, 3.65)	0.398
Q4		3.72 (1.32, 10.47)	0.013
Recurrence risk			
TyG index		0.79 (0.53, 1.17)	0.233
TyG index quartile	Q1		
Q2		0.83 (0.56, 1.23)	0.345
Q3		1.20 (0.80, 1.80)	0.383
Q4		0.81 (0.49, 1.33)	0.400
Adverse outcomes			
TyG index		0.78 (0.52, 1.17)	0.228
TyG index quartile	Q1		
Q2		0.78 (0.52, 1.17)	0.230
Q3		1.18 (0.78, 1.78)	0.436
Q4		0.76 (0.46, 1.27)	0.299

Adjusted age, sex, admission NIHSS score, admission BI score, hypertension, atrial fibrillation, cerebral blood supply artery stenosis, hyperhomocysteinemia, smoking, drinking, sedentary, TC, HDL, LDL, urea nitrogen, creatinine, albumin, globulin, direct bilirubin, indirect bilirubin, ALT, AST, white blood cell count, red blood cell count, platelet count and hemoglobin concentration.

### 3.4 Subgroup analysis of the relationship between TyG index and one-year outcomes

**Figure 2:**
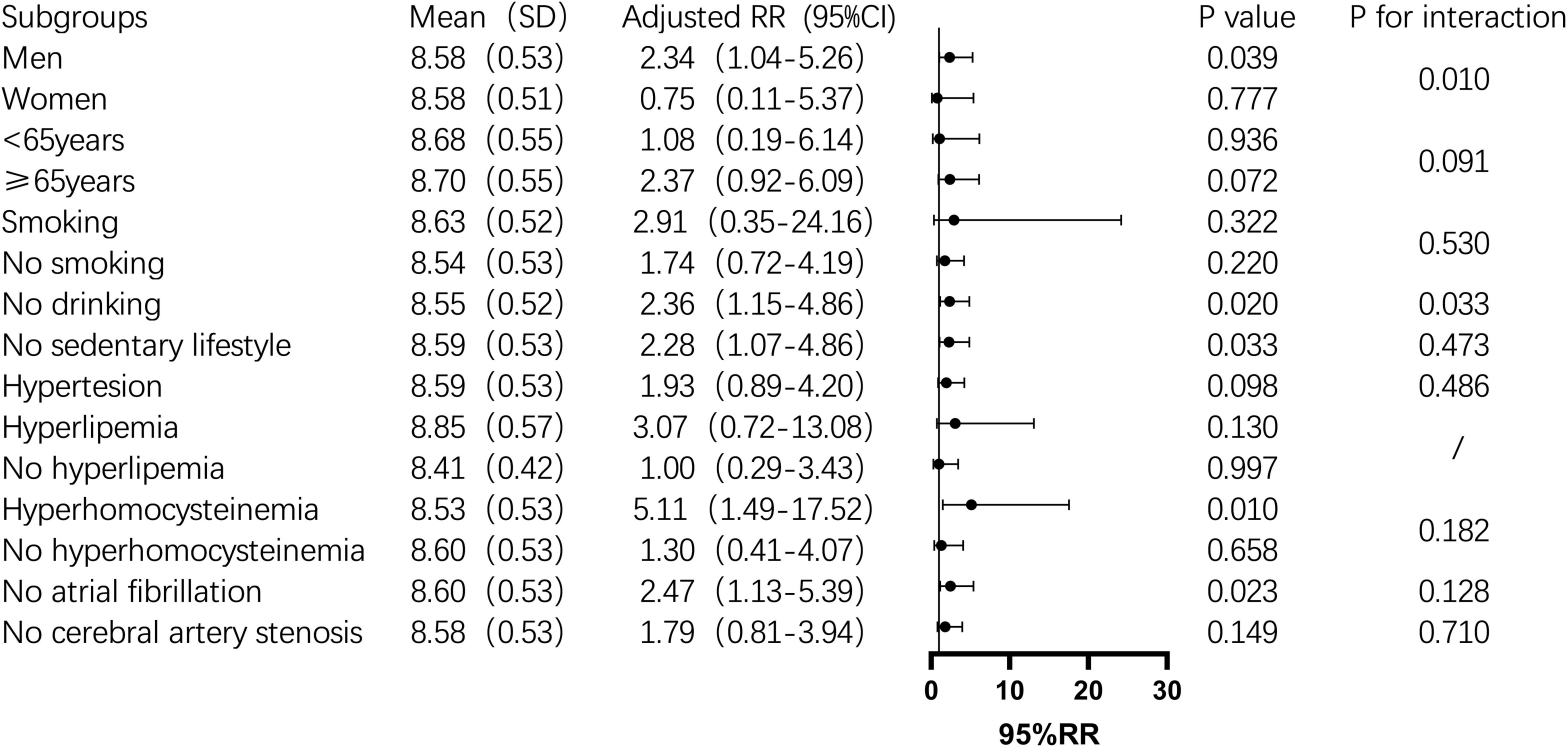
Subgroup analysis of the relationship between TyG index and one-year post-stroke mortality in non-diabetic patients. Image 2 showed that the mean TyG index (standard deviation), adjusted relative risk (95% confidence interval), P value,and interaction P value across different subgroups. Among these, the TyG index was a risk factor for death within one year after stroke in the male, non-smoking, non-sedentary, hyperhomocysteinemia, and non-atrial fibrillation subgroups (P<0.05), while its predictive role was not significant in other subgroups. Additionally, there was an interaction between the TyG index and gender and smoking status, which together influenced the mortality rate one year after stroke (P<0.05).

**Figure 3:**
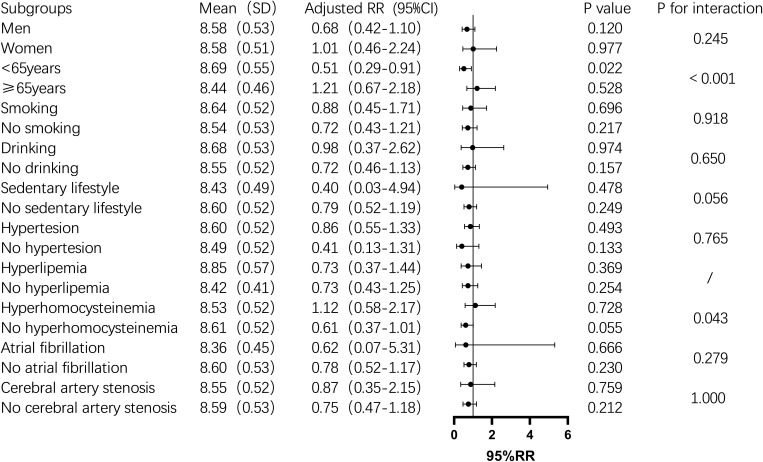
Subgroup analysis of the relationship between TyG index and one-year post-stroke recurrence rate in non-diabetic patients. Image 3 displays the mean TyG index(standard deviation),adjusted relative risk(95% confidence interval), P value, and interaction P value across different subgroups. Among them, for individuals under 65 years of age,an increase of 1 unit in the TyG index was associated with a 49% reduction in the risk of stroke recurrence (P=0.022), and there was an interaction between the TyG index and age group,which together influenced the recurrence of stroke one year after the event in non-diabetic patients(P<0.05).In other subgroups, the impact of the TyG index was not significant. However, the TyG index interacted with age groups and hyperhomocysteinemia status, jointly influencing the recurrence of stroke one year later(P<0.05).

**Figure 4:**
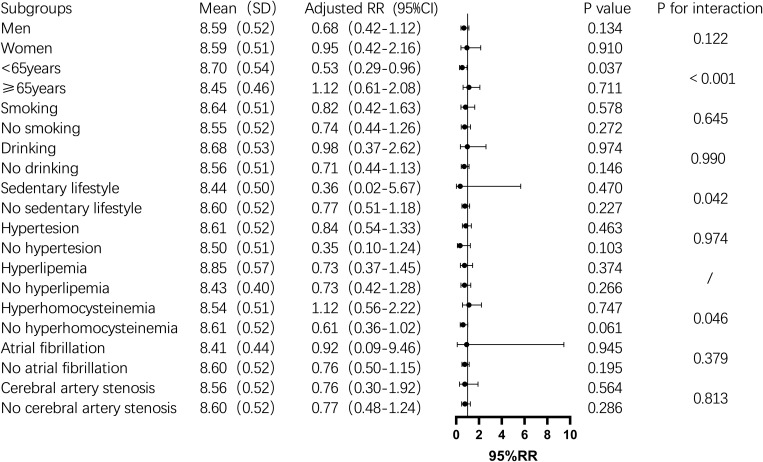
Subgroup analysis of the relationship between TyG index and one-year post-stroke adverse functional outcomes in non-diabetic patients. Image 4 showed the mean TyG index(standard deviation), adjusted relative risk(95% confidence interval), P value,and interaction P value across different subgroups. It was found that among people under 65 years old, for every one-unit increase in the TyG index,the risk of adverse outcomes after stroke increased by 47%(P=0.037), and there was an interaction between age group and TyG index, which together influenced the incidence of adverse outcomes one year after stroke in non-diabetic patients (P<0.001). While the TyG index did not significantly influence adverse outcomes one year after stroke in other subgroups, it interacted with age groups, hyperhomocysteinemia status, and a sedentary lifestyle, collectively affecting the poor functional outcomes (P<0.05).

Subgroup analysis showed that the TyG index was significantly associated with one-year mortality in men, non-drinkers, those with a sedentary lifestyle, hyperhomocysteinemia, and non-atrial fibrillation patients (P < 0.05). In contrast, in the female subgroup, the TyG index was not significantly associated with one-year mortality (P > 0.05). In non-diabetic men, the mortality risk increased by 134% for each unit increase in the TyG index. The risk also increased significantly among non-drinkers (2.36-fold), non-sedentary individuals (2.28-fold), and those with hyperhomocysteinemia (5.11-fold) (**Figure 2**). For stroke recurrence, TyG index was identified as a protective factor in patients under 65 (RR: 0.51, P = 0.022). There was a significant interaction between age and TyG (P < 0.001), as well as between hyperhomocysteinemia and TyG (P = 0.043) in predicting recurrence risk (**Figure 3**). In terms of adverse outcomes, TyG was a protective factor in patients under 65 years old (RR: 0.53, P = 0.037), and interactions between TyG, age, sedentary behavior, and hyperhomocysteinemia significantly influenced outcomes (P < 0.05) (**Figure 4**).

## 4 Discussion

The primary objective of this study was to investigate the association between the TyG index and one-year outcomes—specifically mortality, recurrence, and adverse functional outcomes—among non-diabetic patients with acute IS. Our study revealed that the TyG index were significantly associated with the risk of death one year after stroke, with the highest quartile (Q4) showing a notably increased risk of mortality. Furthermore, subgroup analysis demonstrated that the TyG index was significantly correlated with one-year mortality in specific populations, including men, non-drinkers, those with a non-sedentary lifestyle, individuals with hyperhomocysteinemia, and patients without atrial fibrillation. Although the TyG index did not show a significant association with stroke recurrence or poor outcomes in the overall analysis, subgroup analyses uncovered that the TyG index served as a protective factor against recurrence and adverse outcomes in patients younger than 65. However, it emerged as a risk factor in individuals with hyperhomocysteinemia. Moreover, interactions between the TyG index and both age and hyperhomocysteinemia further influenced the one-year prognosis of stroke patients.

The relationship between the TyG index and the risk of post-stroke mortality has been reported in several prior studies, with higher TyG index consistently associated with increased mortality. A meta-analysis of 18 studies revealed a significant correlation between elevated TyG index and adverse stroke outcomes, including recurrence and mortality (14). Cai et al. found a significant association between the TyG index and both in-hospital and ICU all-cause mortality in IS patients, particularly in non-diabetic, non-atrial fibrillation patients and those under 65 years of age (17). Similarly, Huang et al. reported that higher TyG index was significantly associated with increased all-cause mortality in critically ill hemorrhagic stroke patients, particularly in men and diabetic individuals. Notably, the 30-day mortality was significant, while the 90-day mortality was significant in men and those over 65 years old, with one-year mortality significantly higher in individuals over 65 (18). Moreover, higher TyG index was linked to higher in-hospital mortality, underscoring its role in short-term prognosis, elevated TyG index was associated with an increased risk of all-cause mortality in IS patients (15, 22). Jiang et al., incorporating time variables, demonstrated that the TyG index was negatively correlated with stroke mortality risk, with a significant interaction between age and TyG index in patients over 65, based on the MIMIC-IV database (16). In contrast, in a study of non-diabetic IS patients, the TyG index was significantly associated with increased mortality risk, particularly in the highest quartile of TyG; however, the area under the ROC curve (AUC) was 0.55, indicating limited clinical predictive value (26). Consistent with these findings, our study also found that the TyG index was an independent predictor of one-year mortality in IS patients, even after adjusting for multiple factors. Patients in the highest TyG quartile had a substantially higher risk of stroke-related death compared to those in the lowest quartile. Further subgroup analysis revealed that the TyG index had a stronger association with one-year post-stroke outcomes in men and non-atrial fibrillation patients, in line with the findings of Cai et al. (17). Our study, however, focused on long-term mortality, whereas the use of oral anticoagulants in atrial fibrillation patients may reduce stroke incidence over time, possibly explaining the more pronounced effects observed in non-atrial fibrillation patients (37). Among female patients, the TyG index did not show a significant association with one-year mortality. The smaller sample size in the female subgroup may have limited the statistical power to detect a potential association, and sex-specific differences in metabolic and vascular risk profiles may also contribute to these findings. Further studies with larger female cohorts are needed to clarify the prognostic role of the TyG index in women.

Interestingly, we also discovered that the TyG index was a significant risk factor for mortality among non-drinkers and those without a sedentary lifestyle. Mostofsky et al. noted that moderate alcohol consumption is associated with an immediate increase in cardiovascular risk, which diminishes after 24 hours and may offer protection against ischemic stroke within a week. This protective effect may be absent in non-drinkers, contributing to the higher mortality observed in this group (38). Additionally, sedentary individuals often consume polyphenol-rich foods like chocolate and tea, which have been linked to a reduced risk of stroke and diabetes (39). Since our study focused on non-diabetic IS patients, unrecognized internal mechanisms may account for the pronounced effect of the TyG index in the sedentary population.

Regarding stroke recurrence, prior studies have demonstrated a strong correlation between the TyG index and recurrence risk in various populations. In the general population, the highest quartile of TyG was significantly associated with one-year recurrence in IS patients, with an AUC of 0.719, suggesting improved clinical predictive value (22). The highest TyG quartile was associated with an increased risk of recurrence within one year, particularly in women (25). Among elderly IS patients, higher TyG index was linked to recurrence after one year, especially in the highest quartile (23). In mild IS patients with hypertension and symptomatic intracranial atherosclerosis, a synergistic effect was observed between elevated TyG and recurrence risk within three months (24). However, in non-diabetic AIS patients, although the third and fourth TyG quartiles were independently associated with increased recurrence risk, the AUC was 0.56, indicating limited clinical utility (26). In contrast to previous studies, our univariate and multivariate analyses did not find a significant relationship between the TyG index and stroke recurrence at one year. However, subgroup analysis revealed that the TyG index acted as a protective factor against IS recurrence in patients younger than 65, with a significant interaction between TyG index and age, jointly influencing recurrence risk. While this negative association between TyG and recurrence may initially appear counterintuitive, the previous study have shown that the long-term impact of diabetes mellitus on stroke risk varies by age, with increased risk observed in patients under 65 but not in those older than 65 (40). By focusing on non-diabetic IS patients, we excluded the confounding effects of diabetes on stroke recurrence, which may partly explain the differing results compared to previous studies. Moreover, the long follow-up period and the complex physiological changes during the acute phase of stroke could have influenced the predictive power of the TyG index, leaving certain mechanisms unclear.

There are few studies on the relationship between TyG index and adverse outcomes in stroke survivors. Zhou, Y. et al. found that TyG index was related to the increased risk of neurological deterioration in IS patients after one year (22); Retrospective study found that higher TyG index was associated with higher risk of adverse functional outcome at discharge (15). Contrary to expectations, both the single factor and the multiple factors in this study are related to the poor outcome of stroke one year later. However, in subgroup analysis, it is found that the TyG index is a protective factor of poor outcome and interacts with age and sedentary lifestyle, which may be similar to the results of stroke recurrence and death in this study.

Previously, many studies have confirmed that hyperhomocysteinemia is significantly related to the death and recurrence of stroke (41–43). In particular, in the study of death, recurrence and adverse outcome after one year of stroke, we all found that hyperhomocysteinemia has an important influence, especially in recurrence and adverse outcome, which interacts with TyG index as a predictor of stroke prognosis. Poddar, R. and others summarized the mechanism of neurotoxicity induced by hyperhomocysteinemia (44), which emphasizes the serious influence of hyperhomocysteinemia on stroke. However, the interaction between hyperhomocysteinemia and TyG index is found in both stroke recurrence and death, which can independently predict stroke outcome and is more significant than hyperhomocysteinemia alone.

This study has several limitations. First, as a prospective cohort study, no formal *a priori* sample size calculation or statistical power analysis was performed, potentially affecting the ability to detect smaller effect sizes. Second, only non-diabetic patients were included to minimize confounding. However, this limits the generalizability of the study findings to diabetic populations. Future research should include diabetic patients to fully assess the potential value of the TyG index in a broader clinical population. Third, 789 patients were excluded due to missing data, and 438 patients (23.8%) were lost to follow-up. Despite no significant baseline differences found in supplementary analysis, potential selection bias remains. The lack of a centralized death registry prevented systematic tracking of vital status for lost-to-follow-up patients, possibly underestimating mortality. Future studies should use centralized patient tracking systems and national death databases to improve follow-up completeness and data accuracy. Fourth, outcomes were assessed at a fixed one-year point without continuous event monitoring, which limits time-to-event analysis. Finally, detailed information on medication use (e.g., statins, anticoagulants) was not systematically collected, potentially confounding the associations observed. Despite these limitations, the study provides important evidence that the TyG index is an independent predictor of one-year mortality in non-diabetic acute ischemic stroke patients, highlighting its potential value for risk stratification.

## 5 Conclusion

In this prospective cohort study of non-diabetic patients with acute ischemic stroke, the TyG index was identified as an independent predictor of one-year all-cause mortality, particularly in men, non-atrial fibrillation patients, and those with hyperhomocysteinemia. Although no significant association was observed between the TyG index and stroke recurrence or poor functional outcomes in the entire cohort, subgroup analysis revealed that a higher TyG index was associated with reduced risks of recurrence and poor outcomes in patients under 65 years of age. These findings suggested that, in addition to predicting mortality, the TyG index may have broader prognostic utility, especially in certain patient subgroups. However, the predictive value observed in subgroup analyses needs further validation in large-scale longitudinal studies to elucidate the underlying mechanisms. Overall, the TyG index holds promise as a simple and feasible biomarker for individualized risk stratification in non-diabetic ischemic stroke patients. Integrating it into stroke care could optimize medical resource allocation and reduce the burden of stroke-related mortality and disability.

## Data Availability

The raw data supporting the conclusions of this article will be made available by the authors, without undue reservation.
