# Supplementary material for: Triglyceride-glucose index as a predictor of one-year mortality in non-diabetic acute ischemic stroke
Source: Front Endocrinol (Lausanne). 2025 May 22;16:1523787. doi: 10.3389/fendo.2025.1523787 (PMC12137084; doi:10.3389/fendo.2025.1523787)
Supplement: Supplementary file 1 [file Table1.docx]

Supplemental Table 1. Sensitivity analysis by baseline characteristics between the followed and lost-to-follow-up groups

| Characteristics | Following-up Group | Loss to follow-up Group | P |
| --- | --- | --- | --- |
| Total, n (%) | 1403 (76.2) | 438 (23.8) | — |
| Age, years old | 62.99 (11.70) | 64.19 (11.83) | 0.061 |
| Age group, n (%) |  |  | 0.080 |
| < 45 years old | 66 (4.7) | 15 (3.4) |  |
| 45-64 years old | 703 (50.1) | 210 (47.9) |  |
| 65-79 years old | 520 (37.1) | 161 (36.8) |  |
| ≥80 years old | 114 (8.1) | 52 (11.9) |  |
| Admission NIHSS scores | 5.96 (5.78) | 6.40 (5.62) |  |
| Admission BI scores | 64.96 (29.66) | 62.55 (27.38) |  |
| Hypertension, n (%) |  |  | 0.215 |
| No | 418 (29.8) | 117 (26.7) |  |
| Yes | 958 (70.2) | 321 (73.3) |  |
| Hyperlipidemia, n (%) |  |  | 0.060 |
| No | 1038 (74.0) | 304 (69.4) |  |
| Yes | 365 (26.0) | 134 (30.6) |  |
| Atrial fibrillation, n (%) |  |  | 0.314 |
| No | 1287 (91.7) | 395 (90.2) |  |
| Yes | 116 (8.3) | 43 (9.8) |  |
| Hyperhomocysteinemia, n (%) |  |  | 0.229 |
| No | 1220 (87.0) | 371 (84.7) |  |
| Yes | 183 (13.0) | 67 (15.3) |  |
| Smoking history, n (%) |  |  | 0.169 |
| No | 802 (57.2) | 234 (53.4) |  |
| Yes | 601 (42.8) | 204 (46.6) |  |
| Drinking history, n (%) |  |  | 0.593 |
| No | 1075 (76.6) | 341 (77.9) |  |
| Yes | 328 (23.4) | 97 (22.1) |  |
| Sedentary lifestyle, n (%) |  |  | 0.335 |
| No | 1295 (92.3) | 398 (90.9) |  |
| Yes | 108 (7.7) | 40 (9.1) |  |
| Cerebral artery stenosis, n (%) |  |  | 0.066 |
| No | 1109 (79.0) | 328 (74.9) |  |
| Yes | 294 (21.0) | 110 (25.1) |  |
| TyG index | 8.58 (0.53) | 8.61 (0.48) | 0.238 |
| TyG index quartile |  |  | 0.289 |
| Q1 | 533 (38.0) | 152 (34.7) |  |
| Q2 | 416 (29.7) | 149 (34.0) |  |
| Q3 | 324 (23.1) | 93 (21.2) |  |
| Q4 | 130 (9.3) | 44 (10.0) |  |
